# A case of early-onset periodontitis with vitamin D deficiency: A case report and literature review

**DOI:** 10.1097/MD.0000000000035321

**Published:** 2023-09-29

**Authors:** Chen Li, Jinmei Zhang, Lufei Wang, Jingmei Yang

**Affiliations:** a State Key Laboratory of Oral Disease & National Clinical Research Center for Oral Diseases, Department of Periodontics, West China Hospital of Stomatology, Sichuan University, Chengdu, China; b Guangxi Key Laboratory of the Rehabilitation and Reconstruction for Oral and Maxillofacial Research & Department of Orthodontics, College and Hospital of Stomatology, Guangxi Medical University, Nanning, China.

**Keywords:** case report, immune regulation, periodontitis, vitamin D

## Abstract

**Rationale:**

Periodontitis is an inflammatory disease with multifactorial etiology. Vitamin D, a fat-soluble vitamin, has protective effects on inflammatory response in various systemic conditions. The clinical features of vitamin D deficiency include growth failure, hypotonia, pathologic fractures, rachitic rosary, tetany and so on. Here we present a case of 12-year-old girl affected by early-onset periodontitis accompanied with vitamin D deficiency.

**Patient concerns:**

A 12-year-old girl with gingival redness, bleeding associated with tooth brushing, and mandibular anterior teeth movement, with difficulty in mastication for the past 2 months. There is no relevant family history or special systemic disease history. The serological microelement test showed vitamin D levels were significantly lower than normal range. Immunological test showed abnormal CD4^+^/CD8^+^(CD3^+^CD4^+^/CD3^+^CD8^+^) ratio as well.

**Diagnoses:**

Based on the clinical and serological findings, this patient was ultimately diagnosed with early-onset periodontitis accompanied with vitamin D deficiency.

**Interventions:**

The main treatments for this patient were 3-fold: periodontal therapy, vitamin D supplement and oral hygiene instructions.

**Outcomes:**

Following 1-year therapy, periodontal conditions recovered and became stable. And serological vitamin D levels returned to normal range.

**Lessons:**

The case of interest serves as an important reminder to clinicians, that the early-onset periodontitis may be associated with micronutrients abnormalities, and early-diagnosis and treatment could avoid the body heathy disorders.

## 1. Introduction

Periodontitis is characterized by progressive destruction of the tooth-supporting apparatus, initiated by dysbiosis of plaque biofilms, that eventually results in tooth loss. In populations worldwide, the estimated pooled prevalence for aggressive periodontitis is 1.6% while the periodontal health rate of 12-year-old groups in China is 41.6%.^[1]^ The etiologic and contributory factors of periodontitis, especially in adolescents, are complex, consisting of genetic components,^[2]^ bacterial components,^[3]^ smoking, and any other systemic conditions.^[4]^ Systemic disorders, such as micronutrients abnormalities, could modify the host response to bacterial challenge and result in greater susceptibility to periodontitis.^[4]^ Vitamin D, an indispensable element in calcium homeostasis and immune function,^[5]^ has been proven to be associated with high susceptibility to oral diseases, like caries^[6]^ and periodontal disease.^[7]^ Several cross-sectional trials have found that vitamin D deficiency may be a risk factor for periodontitis.^[8–14]^ A recent meta-analysis also supports this conclusion.^[15]^ Up to now, many studies about the relationship between vitamin D deficiency and periodontitis have been reported in adults, while few study reported the association between vitamin D deficiency and periodontitis in juvenile.^[16]^

Here, we present a case of a 12-year-old girl with severe and rapid progression of periodontal destruction accompanied by vitamin D deficiency which indicates that vitamin D deficiency presents itself as a risk factor of periodontitis, especially in the child. Also, the findings of this case serve as a significant reminder to clinicians. When dental practitioners encounter periodontitis in child, there is a need to increase the awareness of systemic disorders, like vitamin D deficiency, to avoid the adverse effect on physical development.

## 2. Case report

Written informed consent was obtained from the patient and her parents for inclusion in this report.

A 12-year-old girl was referred to the Department of Periodontics, West China Hospital of Stomatology, Sichuan University, with the chief complaint of gingival redness, bleeding associated with tooth brushing, and mandibular anterior teeth movement, with difficulty in mastication for the past 2 months.

**Figure 1.**
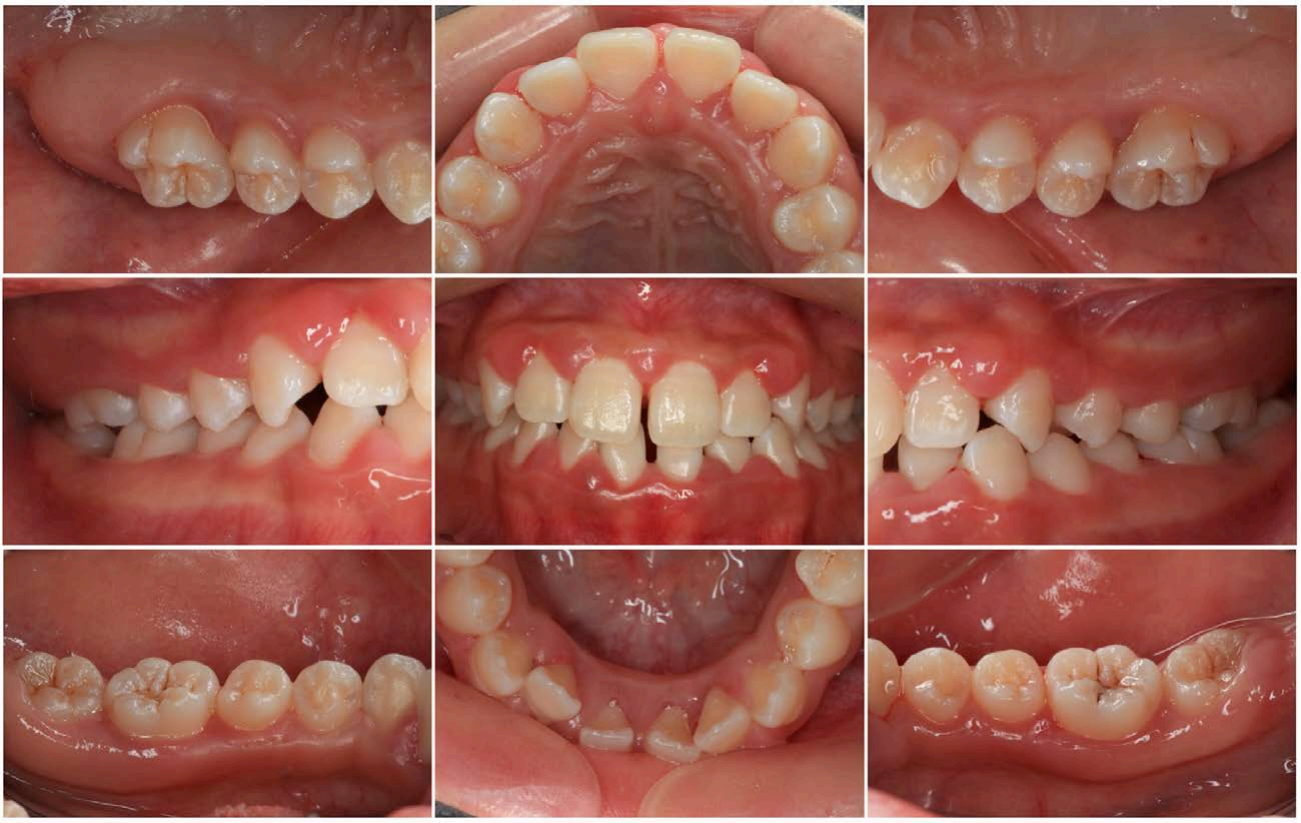
Intraoral view of the patient at the first appointment.

**Figure 2.**
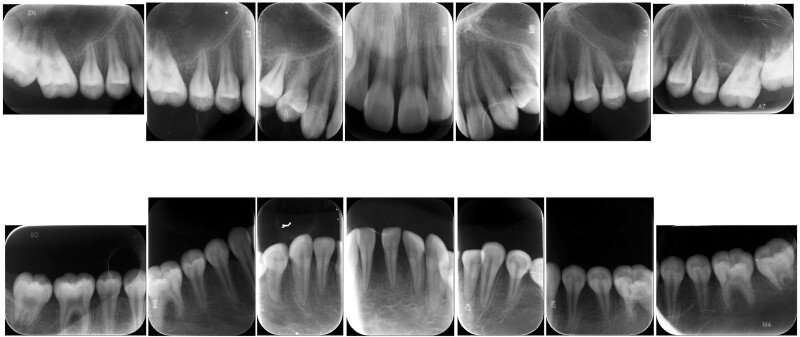
Periapical radiographs showing alveolar bone loss at the first molars and incisors.

Her past medical history was noncontributory, and there is no relevant family history or special systemic disease history. On intraoral examination (Fig. 1), the patient had a Class III malocclusion with a space between the mandibular central incisors, presenting with grade II mobility. Her gingiva was red and tender, bleeding when probed, and her interdental papilla was blunted. Radiographic examination (Fig. 2) revealed alveolar bone loss localized at the first molars and incisors. No significant abnormalities were found in further clinical oral examinations. The serological microelement test showed vitamin D levels were deficient at 8.2 ng/mL (normal range is between 30–100 ng/mL). The serological concentration of Ca^2+^ is 1.48 mmol/L (normal range is between 1.42–2.01 mmol/L), which is relatively low. The cluster of differentiation CD4^+^/CD8^+^ (CD3^+^CD4^+^/CD3^+^CD8^+^) ratio was low at 0.7 (normal range is between 1.5–2.0) while thyroid function tests were normal. In addition, the patient’s bone density report showed a *Z* score of −0.74, which was in the normal range (−1, +1) but close to the critical load.

The provisional diagnosis was the localized aggressive periodontitis (1999 classification)^[17]^ and periodontitis (Stage III, localized, Grade C) (2018 classification).^[18]^

The treatment plan for this patient was periodontal therapy combined with a vitamin D supplement (2000 U q.d po). Periodontal treatment included scaling and root planing combined with systemic administration of 500 mg amoxicillin and 250 mg metronidazole thrice daily for 7 days (the patient weighs 41.5 kg). Oral hygiene instructions were also emphasized throughout the treatment process. In addition to medication, dietary guidelines were provided to the patient and her parents to encourage a higher intake of vitamin D rich foods.

**Figure 3.**
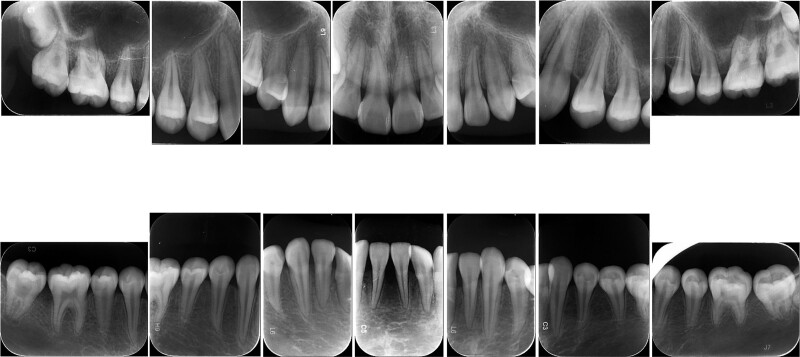
Periapical radiographs showing the formation of the bone-white line.

**Figure 4.**
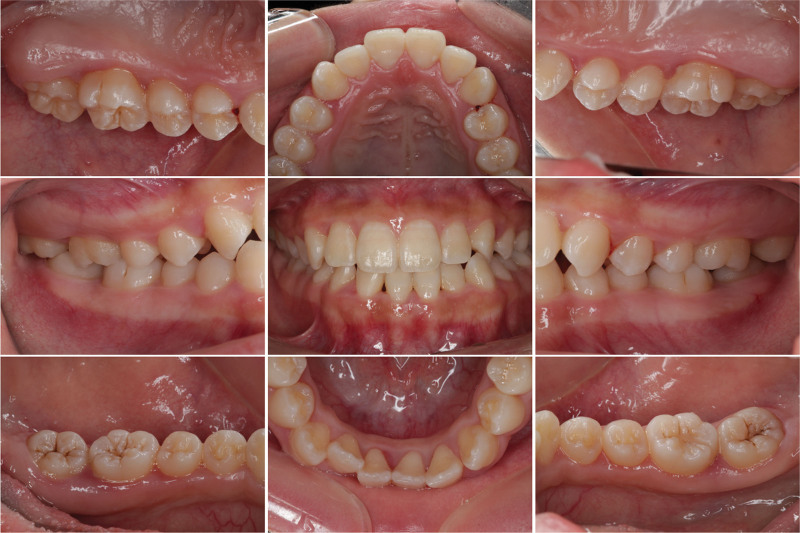
Intraoral view of the patient 1 year later.

After initial periodontal therapy, the patient came to the hospital for a follow-up every 3 months and presented some residual periodontal pockets. One year later, the serological vitamin D normalized at 32.5 ng/mL (normal range 30–100 ng/mL) and the periodontal condition became stable, as indicated by formation of the bone-white line (Fig. 3). However, the patient’s oral hygiene was not very satisfactory. As dental plaque was visible on the teeth surface, and the interdental papillae were slightly red (Fig. 4).

## 3. Discussion

The etiology and underlying risks of periodontitis have remained controversial, especially in adolescents.^[19]^ Despite extensive efforts over the last few decades, the exact pathogenesis of periodontitis in adolescence has not yet been clarified. Polymorphisms of BRAF V600E and TNSALP, autosomal genetic variation and some other syndromes are common factors for early-onset periodontitis (Table 1).^[20–29]^ Nonetheless, we commonly ignore the roles of nutrition in the onset and development of periodontitis. In this case, the patient’s systemic examination report showed deficient vitamin D levels with no other abnormalities. Additionally, the serological test only reported abnormal expression of vitamin D levels with the cluster of differentiation CD4^+^/CD8^+^ (CD3^+^CD4^+^/CD3^+^CD8^+^) ratio displaying half of the average index, which was potential evidence that vitamin D deficiency may be a risk factor that promotes periodontitis progression.

**Table 1:** Case reports showing early-onset periodontitis accompanied by some syndromes.

Author	Year	Disease	Age	Intraoral characteristics	Systemic characteristics	Dentition	Etiology
Qi^[16]^	2021	Vitamin D deficiency	5	Movement and loss of partial teeth and deep periodontal pockets of remaining teeth	Low vitamin D level	Deciduous	Vitamin D receptor gene mutant
Stock^[24]^	2021	Ehlers-Danlos Syndrome	5	Severe gingival recession and significant alveolar bone loss	Joint hypermobility, skin hyperextensibility and connective tissue fragility	Deciduous	Autosomal dominant genetic
Chew^[26]^	2020	Maturity onset diabetes	21	Multiple periodontal abscesses and severe dental fluorosis	Renal cyst and diabetes syndrome	Permanent	Single nucleotide polymorphisms
Fonseca^[25]^	2020	Human immunodeficiency virus	28	Gingival bleeding, necrosis and ulceration	Constant diarrhea, vomiting, flu, and progressive weight loss	Permanent	Human immunodeficiency virus infection
Panis^[20]^	2016	Langerhans cell histiocytosis	21	Recurrent episodes of dull pain in the gingiva	Gum swelling, periosseous abscess and decreased vision	Permanent	BRAF V600E mutant
Iqtadar^[28]^	2015	Papillon-Lefevre syndrome	16	Atrophy of the alveolar bone	Palmoplantar keratoderma and pyogenic infections	Permanent	Autosomal recessive genetic
Chen^[27]^	2013	Neutropenia	8	Recurrent oral ulcers	Pharyngitis and otitis media	Mixed	Congenital
Khocht^[22]^	2010	Chediak-Higashi syndrome	13	Swollen gingival tissues	Bleeding and multiple infections	Permanent	Autosomal recessive genetic
Sasak^[23]^	2004	Down’s syndrome	18	Intense mobility of partial tooth and local gingival swelling	Intellectual disability	Permanent	Trisomy of the chromosome 21
Watanabe^[21]^	1999	Rathbun syndrome	25	Tooth loss and severe periodontal destruction	Hypophosphatasia and skeletal mineralization defect	Permanent	TNSALP gene mutant
Kono^[29]^	1997	Hemophagocytic syndrome	16	Slight gingival inflammation and moderate bone loss	Chronic fever and splenomegaly	Permanent	Autosomal recessive genetic

**Figure 5.**
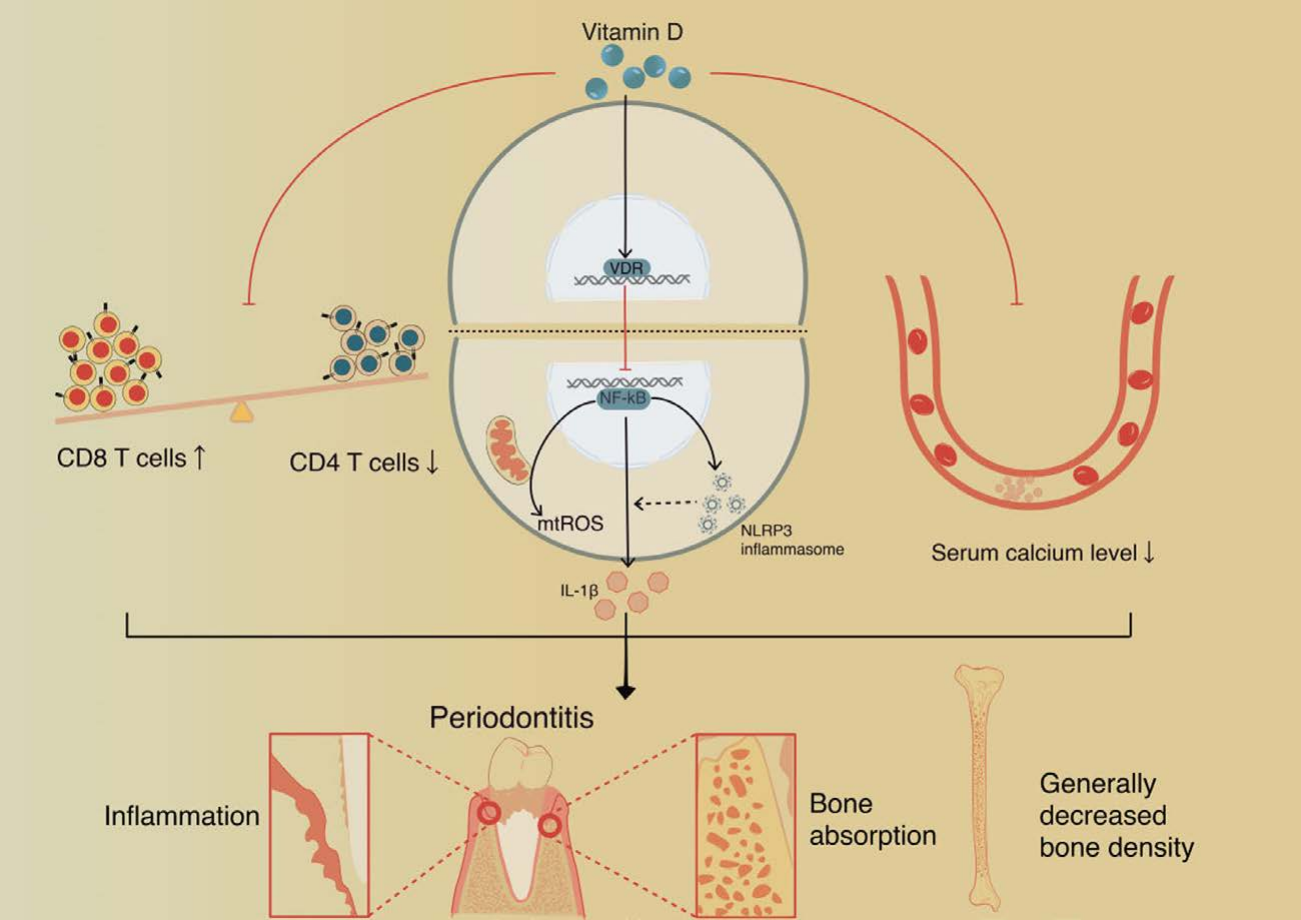
Mechanism figure showing potential pathways by which vitamin D impacts on periodontitis in this case. IL-1β =interleukin-1β, mtROS = mitochondria reactive oxygen species, NLRP3 =nucleotide-binding domain-like receptor family, pyrin domain-containing protein 3.

Vitamin D, a fat-soluble biomolecule, is intimately involved in a wide variety of biological processes, including calcium metabolism, cell proliferation, cell differentiation, and bone preservation.^[30]^ A recently published systematic review and meta-analysis also proved that circulating vitamin D levels were significantly lower in periodontitis patients compared to a healthy control.^[15]^ In a large prospective study with 20 years of follow-up, low levels of vitamin D were associated with a greater risk of tooth loss along with periodontitis.^[31]^ Several cross-sectional observational studies have revealed the association between vitamin D deficiency and periodontitis, and suggested that supplementation with vitamin D may help to maintain periodontal health.^[7]^ There are feasibly several pathways that vitamin D deficiency contributes to occurrence of early-onset periodontitis in this case (Fig. 5).

Vitamin D could prevent the progression of periodontal disease through antimicrobial and anti-inflammatory responses. A previous study proved that vitamin D could stimulate the release of antimicrobial peptides, such as defensins,^[32]^ to help to kill the periodontal pathogen, like *Actinobacillus actinomycetemcomitans*. Grenier et al^[33]^ found that vitamin D decreases the number of *Porphyromonas gingivalis* and its expression of genes related to host colonization and tissue destruction such as *fimA, hagA, hagB, rgpA, rgpB*, and *kgp*. Evidence suggests that vitamin D is anti-inflammatory through inhibition of the NF-κB/NLRP3 signaling pathway.^[34]^ Subsequent IL-1β and reactive oxygen species decrease to hinder the progression of periodontitis.^[34]^

The deficiency of vitamin D would contribute to the CD4^+^/CD8^+^ reduction, which could reflect cellular immune dysfunction. The ratio of CD4^+^ (helper or regulatory CD4^+^ T cells)/CD8^+^ (cytotoxic CD8^+^ T cells) is an important indicator, reflecting the activity of T lymphocytes as well as the body’s overall immune function and disease development. Thus, maintaining the proper number and proportion of CD4^+^ and CD8^+^ T cells is essential for optimal host defense. In our case, the patient’s CD4^+^/CD8^+^ (CD3^+^CD4^+^/CD3^+^CD8^+^) ratios were low, indicating that the patient’s ability to defend against infection was impaired, and the risk of periodontitis increased. Our findings are consistent with previous reports by Kinane et al^[35]^ and Handono et al.^[36]^ Ding et al^[37]^ found that 1,25(OH)2D3 could elevate CD4^+^/CD8^+^ T lymphocytes, reduce CD8^+^ T lymphocytes, and reduce expressions of inflammatory factors. The animal study proved that the supplement of vitamin D could significantly increase the ratios of CD4^+^/CD8^+^ in mice.^[38]^ Therefore, vitamin D deficiency may result in decreased CD4^+^/CD8^+^, reducing the ability of the host to prevent an exaggerated immune response to pathogenic microorganisms that may negatively impact host’s immune function and cause the progression of periodontitis.

In addition, vitamin D is a biologically active metabolite, and it could maintain the homeostasis of calcium and phosphate in blood. In this case, the level of Ca^2+^ was relatively low, which was possibly contributed by the deficiency of vitamin D in the patient. As a result, this decreased level of Ca^2+^ could destroy bone remodeling and mass and reduce bone mineral density that is followed by osteoporosis or alveolar bone resorption. That could explain the reason why the patient’s bone density (*Z* score) was −0.74. Although this Z score was in the normal range, it was close to the critical load. The decreased mass and fragility of the alveolar bone were prone to facilitate oral biofilm infection, spell host immunological responses, and accelerate the progression of periodontitis.^[39]^

In our case, vitamin D supplementation proved to be useful in treating periodontitis adjunctively, which is consistent with the studies reported before.^[16,40]^ With the combination of scaling and root planing and vitamin D supplementation, the periodontal condition has improved. This case reported an early-onset periodontitis accompanied by vitamin D deficiency in adolescence, and ulteriorly elaborated on inversion of CD4^+^/CD8^+^ ratio as well as the decreased bone density brought by vitamin D deficiency.

Except for vitamin D, the deficiency of vitamin C and vitamin E, as typical antioxidants, is also a potential risk for preventing periodontitis. Kuzmanova et al^[41]^ found that the plasma vitamin C in patients with periodontitis was lower compared to healthy controls. Vitamin C concentration was inversely proportional to periodontal pocket depth, and patients with lower vitamin C level presented higher disease stage of periodontitis.^[42]^ Additionally, intake of vitamin C could significantly suppress the gingival bleeding in patients with periodontitis.^[43]^ In regards to vitamin E, a large cross-sectional study of US adults showed that serum tocopherol (a major group of vitamin E) levels were inversely associated with the severity of periodontitis.^[44]^ Singh et al^[45]^ found that supplementation of vitamin E improved serum and saliva superoxide dismutase activity, and the periodontal conditions were improved in patients with periodontitis. In a nutshell, micronutrients inclusive of specific vitamin are warranted for periodontal health.

## 4. Conclusion

Systemic risk factors underlying periodontitis are still unclear, especially in the youth. This case report shows that low levels of vitamin D may contribute to the susceptibility of periodontitis. Early diagnosis of these conditions is necessary to prevent periodontal destruction and systemic disorders that vitamin D deficiency may bring.

## Author contributions

**Funding acquisition:** Jingmei Yang.

**Investigation:** Jinmei Zhang.

**Supervision:** Jingmei Yang.

**Writing – original draft:** Chen Li.

**Writing – review & editing:** Jinmei Zhang, Lufei Wang, Jingmei Yang.
